# Supplementary material for: The juxtamembrane linker of synaptotagmin 1 regulates Ca2+ binding via liquid-liquid phase separation
Source: bioRxiv. 2023 Aug 12:2023.08.11.551903. Preprint. [Version 1] doi: 10.1101/2023.08.11.551903 (PMC10441399; doi:10.1101/2023.08.11.551903)
Supplement: Supplementary file 1 [file media-1.docx]

**Supplementary Material**

**The juxtamembrane linker of synaptotagmin 1 regulates Ca**^**2+**^ **binding via liquid-liquid phase separation**

Nikunj Mehta^1^, Sayantan Mondal^2^, Emma T. Watson^1^, Qiang Cui^2^, and Edwin R. Chapman^1^*

^1^Howard Hughes Medical Institute, Department of Neuroscience, University of Wisconsin−Madison, Madison, WI 53705, United States.

^2^Department of Chemistry, Boston University, Boston, MA 02215, United States.

*Corresponding author: Edwin R. Chapman

Email: [chapman@wisc.edu](mailto:chapman@wisc.edu)

This PDF file includes:

	Supplementary Figures 1-7

	Supplementary Tables 1-3

	Supplementary Movies 1,2

**Supplementary Figures**

**Supplementary Fig. 1.** **Conservation of lysine residues in the syt1 juxtamembrane linker**. 

**a, b** Sequences of the juxtamembrane linkers (residues 80-142) from WT (*Rattus norvegicus*) and JuxtaK mutant, in which the lysine residues have been substituted with polar residues. **c** Alignment of all lysine residues in the juxtamembrane linkers of syt1 from multiple species, revealing strong conservation, except in flies. At the bottom, the amino acids used to substitute all but one lysine residue are shown. Aligned using ClustalOmega^1^. 

**Supplementary Fig. 2. Atomistic MD simulations predict multiple hydrogen bonds are formed by lysine residue sidechains within amino acid segment 80-95.**

Snapshots from atomistic simulations of the syt1 linker condensate using CHARMM36 force-field^2^. **a** The condensate at atomistic resolution. **b** Magnified view from the interior of the condensate where a complex hydrogen bond (HB) network is shown to involve the sidechains of two Lys, one Asp, and one Glu residue. **c** Another magnified view of the interior of the condensate, where a single Lys sidechain forms simultaneous HBs with Asp and Glu sidechains. These residues are from different copies of the syt1 linker.

**Supplementary Fig. 3. Syt1 C2AB (80-421)-GFP and GFP-syt1 (80-142) form large oligomers at physiological ionic strength.** 

Representative dynamic light scattering (DLS) frequency distributions of the proteins (**Fig. 3a,b). Supplementary Table 2** shows the average diameters; n = 3. The buffer was 25 mM Tris-HCl (pH 7.4), 100 mM NaCl with 3% PEG 8000. Note: these DLS measurements are not accurate at sizes > 1 µm.

**Supplementary Fig. 4.** **Droplet size correlates with syt1 protein concentration.** 

Syt1 C2AB (80-421)-GFP LLPS was monitored at the indicated protein concentrations. At increasing [protein], there is an increase in droplet size. We note that the number of droplets was confounded by the ability of droplets to fuse with each other, so this parameter was not further analyzed. The buffer was 25 mM Tris-HCl (pH 7.4), 100 mM NaCl, 3% PEG 8000; n = 3.

**Supplementary Fig. 5.** **Truncation and mutation of the juxtamembrane linker in syt1 C2AB as well as the isolated JuxtaK mutant linker, fail to form droplets.** 

Syt1 C2AB-GFP with a truncated or mutated juxtamembrane linker, as well as the isolated JuxtaK linker, with and without 3% PEG 8000 were monitored for droplet formation, over the indicated [protein] range. White squares indicate that droplets were not observed under any condition tested; n = 3.

**Supplementary Fig. 6.** **MD simulations predict dissolution of syt1 juxtamembrane linker LLPS droplets at high ionic strength.**

**a** MD simulation of droplet formation by the isolated syt1 (80-142) juxtamembrane linker in 100 mM NaCl after 5 µs of simulation. **b** Droplet partially dissolves at high ionic strength (400 mM NaCl), as determined using the same reparametrized MARTINI v3.0 force field (Scale bars, 4 nm). The color coding is the same as described in Fig. 2c. **c** The distribution of the gain/loss in the number of contacts (ΔQ) in the syt (80-142) juxtamembrane linker compared to the same number of isolated syt1 linkers. The distribution shifts left at 400 mM [NaCl], indicating a loss in the number of pairwise contacts. **d** DLS analysis reveals the dissolution of syt1 C2AB (80-421)-GFP droplets when the [NaCl] is increased from 100 to 400 mM in 25 mM Tris-HCl (pH 7.4) with 3% PEG 8000. **Supplementary Table 3** shows average diameters and number of particles; n = 3.

**Supplementary Fig. 7. Model describing how syt1 LLPS might affect syt1 function.** 

The lysine-rich motif in the syt1 juxtamembrane linker mediates LLPS (Fig. 3c,d, 6a,c), and substitution of these lysine residues disrupts robust, synchronized neurotransmitter release^3^. LLPS reduces the Ca^2+^ sensitivity of syt1, and Ca^2+^ promotes droplet formation. Syt1 LLPS might also favor (-) curvature to facilitate membrane fusion^4^. The juxtamembrane linker harbors sites for post-translational modifications (PTMs) that might regulate LLPS and hence syt1 function. Solid lines represent experimentally established observations; dashed lines indicate proposed phenomenon that are currently under study.

**Supplementary Tables**

|  | DH (cal/mol) | DS (cal/mol/K) | DG (kcal/mol) |
| --- | --- | --- | --- |
| syt1 C2AB (80-421) | DH_1_ = 225 ± 29 | DS_1_ = 19.5 ± 1.2 | DG_1_ = -5.59 ± 0.16 |
|  | DH_2_ = 559 ± 103 | DS_2_ = 18.9 ± 1.3 | DG_2_ = -5.07 ± 0.15 |
|  | DH_3_ = 549 ± 94 | DS_3_ = 17.9 ± 0.3 | DG_3_ = -4.79 ± 0.17 |
|  | DH_4_ = 867 ± 102 | DS_4_ = 16.1 ± 0.7 | DG_4_ = -3.93 ± 0.20 |
|  | DH_5_ = 1040 ± 142 | DS_5_ = 15.1 ± 0.9 | DG_5_ = -3.46 ± 0.23 |
| syt1 C2AB (96-421) | DH_1_ = 96 ± 8 | DS_1_ = 21.5 ± 0.4 | DG_1_ = -6.31 ± 0.02 |
|  | DH_2_ = 618 ± 89 | DS_2_ = 18.9 ± 1.4 | DG_2_ = -5.01 ± 0.14 |
|  | DH_3_ = 661 ± 59 | DS_3_ = 18.7 ± 1.2 | DG_3_ = -4.91 ± 0.09 |
|  | DH_4_ = 832 ± 104 | DS_4_ = 18.1 ± 2.4 | DG_4_ = -4.56 ± 0.03 |
|  | DH_5_ = 1789 ± 102 | DS_5_ = 17.9 ± 2.1 | DG_5_ = -3.55 ± 0.10 |
| syt1 C2AB (80 JuxtaK-421) | DH_1_ = -28 ± 3.1 | DS_1_ = 22.1 ± 1.0 | DG_1_ = -6.61 ± 0.01 |
|  | DH_2_ = -244 ± 51 | DS_2_ = 20.3 ± 0.5 | DG_2_ = -6.29 ± 0.21 |
|  | DH_3_ = -869 ± 256 | DS_3_ = 14.5 ± 1.6 | DG_3_ = -5.19 ± 0.31 |
|  | DH_4_ = -292 ± 101 | DS_4_ = 12.9 ± 1.8 | DG_4_ = -4.14 ± 0.01 |

**Supplementary Table 1**

Thermodynamic properties (G, Gibbs free energy; S, entropy; H, enthalpy) of Ca^2+^ binding to the indicated syt1 cytosolic domain constructs measured using ITC. Data are mean values ± SEM; n = 3.

| Diameter (nm) | syt1 C2AB (80-421)-GFP | syt1 C2AB (142-421)-GFP | syt1 C2AB (80 JuxtaK-421)-GFP | GFP-syt1 (80-142) | GFP-syt1 (80 JuxtaK-142) | GFP |
| --- | --- | --- | --- | --- | --- | --- |
| D1 | 8.8 ± 0.3 (23%) | 8.1 ± 0.6 | 8.2 ± 0.2 | 6.8 ± 0.3 (21%) | 5.8 ± 0.4 | 5.2 ± 0.1 |
| D2 | 1170 ± 91 (67%) |  |  | 891 ± 29 (79%) |  |  |

**Supplementary Table 2**

Table showing the results of the DLS measurements of the syt1 constructs from **Supplementary Fig. 3.** The average diameter of syt1 proteins/oligomers in solution and fraction (%) of small (D1) and large (D2) particles, when two populations were observed; n = 3, error bars represent SEM.

| Diameter (nm) | syt1 C2AB (80-421)-GFP |  |
| --- | --- | --- |
|  | 100 mM NaCl | 400 mM NaCl |
| D1 | 8.8 ± 0.3 (23%) | 8.3 ± 0.4 (96%) |
| D2 | 1170 ± 91 (67%) | 1100 ± 75 (4%) |

**Supplementary Table 3**

DLS measurements of syt1 C2AB (80-421)-GFP at low and high ionic strengths from **Supplementary Fig. 6d**. The average diameter of syt1 proteins/oligomers and fraction of particles (%) at 100 and 400 mM NaCl in 25 mM Tris-HCl (pH 7.4) with 3% PEG 8000; n = 3, error bars represent SEM. 

**Supplementary Movies**

**Supplementary Movie 1.** Fusion of droplets formed by syt1 C2AB (80-421)-GFP (10 µM) in 25 mM Tris-HCl (pH 7.4), 100 mM NaCl, with 3% PEG 8000. See Fig. 4a for a time series of these images. (Scale bars, 2 µm) 

**Supplementary Movie 2.** Photobleaching followed by fluorescence recovery of a circular area within a droplet formed by syt1 C2AB (80-421)-GFP (10 µM) at 25 mM Tris-HCl (pH 7.4), 100 mM NaCl, with 3% PEG 8000. See Fig. 4b for a time series of these images. (Scale bars, 2 µm)

	

**References**

1.	Sievers, F. *et al.* Fast, scalable generation of high‐quality protein multiple sequence alignments using Clustal Omega. *Mol. Syst. Biol.* **7**, 539 (2011).

2.	Huang, J. *et al.* CHARMM36m: an improved force field for folded and intrinsically disordered proteins. *Nat. Methods* **14**, 71–73 (2017).

3.	Courtney, K. C. *et al.* Synaptotagmin 1 oligomerization via the juxtamembrane linker regulates spontaneous and evoked neurotransmitter release. *Proc. Natl. Acad. Sci.* **118**, e2113859118 (2021).

4.	Hui, E., Johnson, C. P., Yao, J., Dunning, F. M. & Chapman, E. R. Synaptotagmin-Mediated Bending of the Target Membrane Is a Critical Step in Ca^2+^-Regulated Fusion. *Cell* **138**, 709–721 (2009).
